# Active site formation mechanism of carbon-based oxygen reduction catalysts derived from a hyperbranched iron phthalocyanine polymer

**DOI:** 10.1186/s11671-015-0881-8

**Published:** 2015-04-14

**Authors:** Yusuke Hiraike, Makoto Saito, Hideharu Niwa, Masaki Kobayashi, Yoshihisa Harada, Masaharu Oshima, Jaehong Kim, Yuta Nabae, Masa-aki Kakimoto

**Affiliations:** Department of Applied Chemistry, The University of Tokyo, 7-3-1 Hongo, Bunkyo-ku, Tokyo, 113-8656 Japan; Institute for Solid State Physics, The University of Tokyo, 5-1-5 Kashiwanoha, Kashiwa, Chiba 277-8581 Japan; Synchrotron Radiation Research Organization, The University of Tokyo, SPring-8, 1-1-1, Koto, Sayo-cho, Sayo-gun, Hyogo, 679-5198 Japan; Department of Organic and Polymeric Materials, Tokyo Institute of Technology, 2-12-1, O-okayama, Meguro-ku, Tokyo, 152-8552 Japan; Current address: Toray Industries, Incorporated, Nihonbashi-Muromachi 2-chome, Tokyo, Japan; Current address: Toyota Motor Corporation, 1200, Mishuku, Susono, Shizuoka, 410-1107 Japan; Current address: Institute of Materials Structure Science, High Energy Accelerator Research Organization (KEK), 1-1 Oho, Tsukuba, Ibaraki, 305-0801 Japan; Current address: OLED R&D Center, Samsung Mobile Display Co., Ltd., Yongin, Gyeonggi 446-811 Korea

**Keywords:** Oxygen reduction reaction (ORR), X-ray photoemission spectroscopy, Electronic structure, Hyperbranched polymer, Iron phthalocyanine (FePc)

## Abstract

Carbon-based cathode catalysts derived from a hyperbranched iron phthalocyanine polymer (HB-FePc) were characterized, and their active-site formation mechanism was studied by synchrotron-based spectroscopy. The properties of the HB-FePc catalyst are compared with those of a catalyst with high oxygen reduction reaction (ORR) activity synthesized from a mixture of iron phthalocyanine and phenolic resin (FePc/PhRs). Electrochemical measurements demonstrate that the HB-FePc catalyst does not lose its ORR activity up to 900°C, whereas that of the FePc/PhRs catalyst decreases above 700°C. Hard X-ray photoemission spectra reveal that the HB-FePc catalysts retain more nitrogen components than the FePc/PhRs catalysts between pyrolysis temperatures of 600°C and 800°C. This is because the linked structure of the HB-FePc precursor has high thermostability against nitrogen desorption. Consequently, effective doping of active nitrogen species into the *sp*^*2*^ carbon network of the HB-FePc catalysts may occur up to 900°C.

## Background

The polymer electrolyte fuel cell (PEFC) is an efficient and clean energy conversion system that produces electricity from the redox reaction of H_2_ and O_2_ gases [1]. Because PEFCs operate at lower temperatures (approximately 80°C) than other types of fuel cells and the oxygen reduction reaction (ORR) at the cathode has sluggish kinetics, Pt is usually used as a catalyst to accelerate the ORR in PEFCs. However, the high cost and poor durability of Pt currently limit the commercialization of PEFCs [2]. Among studies on Pt-free cathode catalysts, carbon-based catalysts have attracted considerable attention as alternatives to Pt catalysts. In particular, carbon-based cathode catalysts synthesized by pyrolyzing transition metal (TM) chelates (such as phthalocyanine or porphyrin complexes) and/or nitrogen precursors [3-19] have demonstrated promising activities. Based on a pioneering study on cobalt phthalocyanine as an oxygen reduction catalyst [3], it has been suggested that nitrogen-coordinated TMs such as TM-N_4_ and TM-N_2_ (TM = Fe, Co) are possible active sites of the ORR in carbon-based cathode catalysts [5-7]. Nitrogen species incorporated into a carbon network, such as pyridine- and pyrrole-like nitrogen [9,10,12,13] and graphite-like nitrogen [11-13,15-17], also contribute to the activity of the ORR [8-17]. In previous studies, we analyzed the electronic structure of carbon-based cathode catalysts derived from mixtures of metallophthalocyanine and phenolic resin (TMPc/PhRs) [17,18]. We found that the residual TM atoms existed mostly as metallic nanoparticles after pyrolysis. It has also been revealed that these TM particles catalyze graphitization by the Yarmulke mechanism [20], serving as centers of graphitization in a manner similar to carbon nanotubes. We have elucidated that nitrogen functionalities in the graphitized carbon network surrounding these TM particles are responsible for the high ORR activity of TMPc/PhRs-derived catalysts [15-17,21]. It is therefore important to introduce more nitrogen into TMPc/PhRs systems to increase their ORR activity. However, there is a trade-off between activity and durability: although graphitization at higher temperature (above 600°C) improves the stability of the catalysts, nitrogen desorption catalyzed by TM atoms and/or desorption of the TM atoms occurs, and this desorption reduces ORR activity [6,13]. Therefore, to obtain catalysts with both high ORR activity and stability, it is essential to develop a process to fabricate highly graphitized carbon-based cathode catalysts with sufficient nitrogen content.

Here we report a novel carbon-based cathode catalyst synthesized from a hyperbranched iron phthalocyanine polymer (HB-FePc). HB-FePc contains numerous branched structures where FePc complexes are connected to each other by a specific linker [22,23]. These HB polymers can solve the trade-off problem mentioned above because they resist thermal decomposition and thus can suppress nitrogen desorption during pyrolysis [23]. In this paper, we explore spectroscopic study on the formation mechanism of ORR active sites in HB-FePc-derived catalysts by comparison of their properties with those of the iron phthalocyanine and phenolic resin (FePc/PhRs)-derived catalysts which do not contain a linker among FePc complexes. We conducted hard X-ray photoemission spectroscopy (HXPES) measurements to elucidate the roles of light elements and residual iron in the ORR. HXPES using synchrotron radiation has many advantages over conventional X-ray photoemission spectroscopy (XPS) because of its high energy resolution and long probing depth. In particular, the average diameter of the iron-centered nanoshell structure in carbon-based cathode catalysts is 20 to 30 nm [14,18,19], which matches the long probing depth of HXPES of over 30 nm well. Our experimental results reveal how the linked structure in the HB-FePc catalysts modifies the thermal process to form ORR active sites and contributes to retain the ORR activity of these catalysts pyrolyzed at temperatures up to 900°C.

## Methods

### Sample preparation and characterization

**Figure 1:**
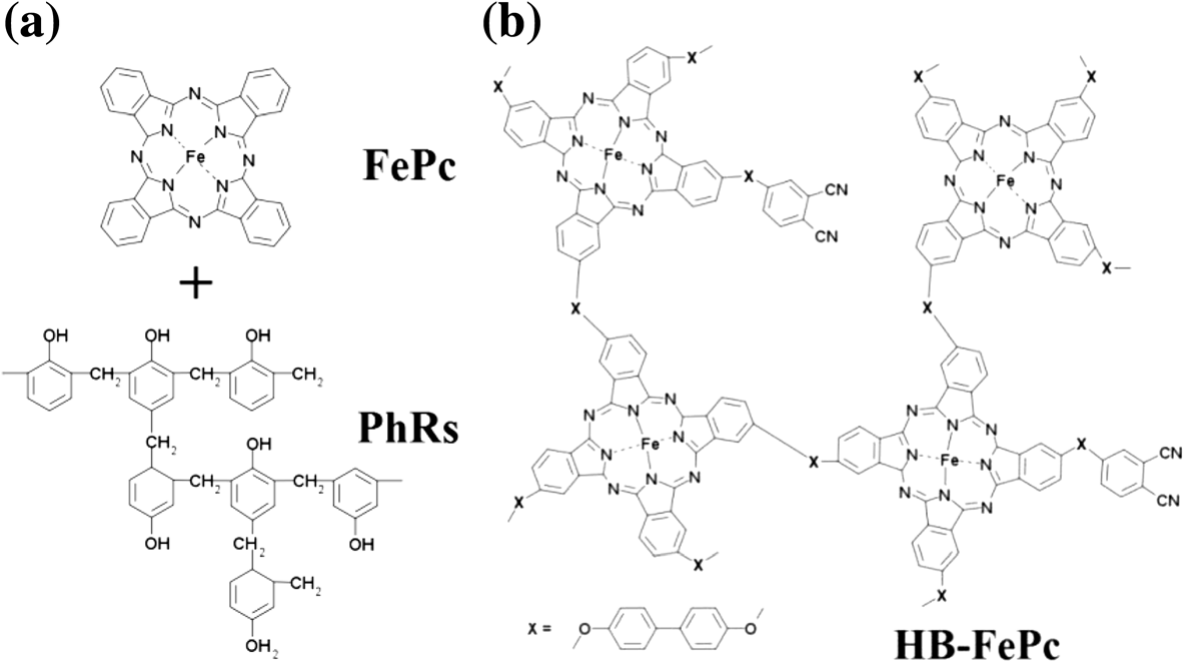
Structural formulae of precursors. **(a)** Iron phthalocyanine (FePc) and phenolic resin (PhRs), and **(b)** hyperbranched iron phthalocyanine polymer (HB-FePc). In this study, biphenyl was used to link HB-FePc (X).

Two different types of carbon-based cathode catalysts were prepared from pyrolysis of (i) a mixture of FePc/PhRs and (ii) HB-FePc with a specific linker (see Figure 1). The Fe content of the HB-FePc precursor was in the range of 2.2 to 2.7 wt% (estimated from thermogravimetry-differential thermal analysis (TG-DTA) measurements), while that of FePc/PhRs was adjusted to 3 wt%. The carbon-based cathode catalysts were synthesized at various temperatures (500°C to 900°C) under N_2_ flow for 5 h. These samples are denoted according to their pyrolysis temperature. For example, Fe600 and HB600 denote FePc/PhRs- and HB-FePc-derived carbon-based cathode catalysts pyrolyzed at 600°C, respectively. A mixture of FePc and PhRs that was not heat treated is denoted FePc/PhRs.

The ORR activities of these samples were evaluated by rotating disk electrode (RDE) voltammetry in 0.5 mol L^−1^ H_2_SO_4_ saturated with oxygen at room temperature. Linear sweep voltammograms were recorded by sweeping the potential from 1.1 to 0 V *vs*. a normal hydrogen electrode (NHE) at 0.5 mV s^−1^, where the working electrode was rotated at a fixed speed of 1,500 rpm. As a measure of the ORR activity, we used the onset potential of the ORR *E*_O2_, which is defined as the voltage at which the reduction current density normalized by the area of a glass-like carbon disk electrode reached −2 μA cm^−2^. The current density at 0.5 V (*vs*. NHE) was also measured as an indicator of catalytic performance.

In order to estimate the specific surface area of the catalysts, N_2_ adsorption measurements were conducted with a volumetric adsorption measurement instrument (Belsorp-mini II, BEL Japan, Inc., Osaka, Japan). X-ray diffraction (XRD) was measured with an X-ray diffractometer (Ultima IV, Rigaku, Tokyo, Japan) using Cu *Kα* radiation operating at 40 kV and 40 mA. TG, DTA, and differential thermogravimetry (DTG) were performed in He gas flow (300 mL min^−1^) using a TG-DTA system (Thermo Plus Evo, Rigaku, Tokyo, Japan) connected to a mass spectrometer (GC-MS-QP2010, Shimadzu, Kyoto, Japan). The reaction section was purged with He gas for 30 min before heating. The temperature was raised from room temperature to 900 K at a rate of 50 K min^−1^, and outlet gas was analyzed by the mass spectrometer.

### HXPES measurements

HXPES measurements were conducted in an ultrahigh-vacuum chamber at room temperature at BL46XU and BL47XU of SPring-8. The photon energy used for the photoemission measurements was 7.94 keV. Insulating FePc/PhRs catalysts Fe500 and Fe550 were mounted on indium sheets to avoid charging problems. In this study, the probing depth of HXPES for the C 1*s*, N 1*s*, O 1*s*, and Fe 2*p* edges is approximately 40 nm, which is about three times larger than the inelastic mean free paths of photoelectrons [24]. HXPES spectra were recorded by an electron analyzer (VG Scienta, R-4000) with a pass energy *E*pass of 200 eV. The overall energy resolution estimated using the Fermi edge of an Au plate was approximately 0.25 eV, and the stability of the absolute energy was within 20 meV. All of the spectra shown in this paper are normalized to the peak height. For detailed chemical analysis, backgrounds of the core level spectra were subtracted by the Shirley method [25]. For the N 1*s* HXPES, each spectrum was fitted with a Voigt function [26], which is a convolution of Gaussian and Lorentzian functions, to decompose it into different chemical species of nitrogen.

## Results and discussion

### Electrochemical and physical properties

**Figure 2:**
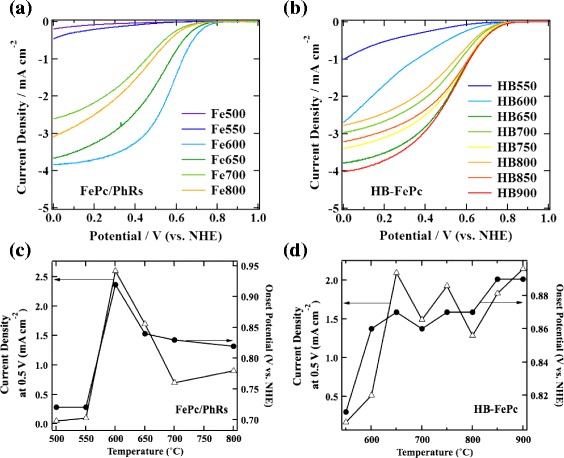
Electrochemical characterization of the catalysts. Linear sweep voltammograms of **(a)** FePh/PhRs and **(b)** HB-FePc catalysts and onset potential and current density plots as a function of pyrolysis temperature (bottom) of **(c)** FePc/PhRs and **(d)** HB-FePc catalysts.

Figure 2 shows linear sweep voltammograms of (a) the FePc/PhRs and (b) HB-FePc carbon-based cathode catalysts (hereafter denoted simply as FePc/PhRs and HB-FePc catalysts) measured by the RDE method. The corresponding onset potential and current density as a function of pyrolysis temperature for the FePc/PhRs and the HB-FePc catalysts are plotted in Figure 2c,d. Among the FePc/PhRs catalysts, Fe600 shows the highest onset potential (0.92 V) with the largest current density at 0.5 V. For pyrolysis temperatures above 600°C, the onset potential decreases slightly and the current density at 0.5 V degrades markedly. In contrast, the ORR activity of the HB-FePc catalysts increases at a pyrolysis temperature of around 650°C (onset potential = 0.87 V for HB650) and remains almost unchanged up to 900°C (0.89 V for HB900). It is remarkable that the ORR activity of the HB-FePc catalysts is retained at such high pyrolysis temperatures compared with that of the FePc/PhRs catalysts. Specific BET surface areas of both samples are summarized in Table 1. The values are between 337 to 564 m^2^ g^−1^, suggesting that the pore structures of these samples do not have significant impacts on the ORR activity.

**Table 1:** **Specific BET surface area of FePc/PhRs and HB-FePc catalysts calculated from nitrogen adsorption measurements**

**Temperature (°C)**	**FePc/PhRs** ^**a**^ **(m** ^**2**^ **g** ^**−1**^ **)**	**HB-FePc (m** ^**2**^ **g** ^**−1**^ **)**
900	-	409
800	407	485
700	444	409
650	437	384
600	515	337
550	506	564

^a^Data from ref. [14].

**Figure 3:**
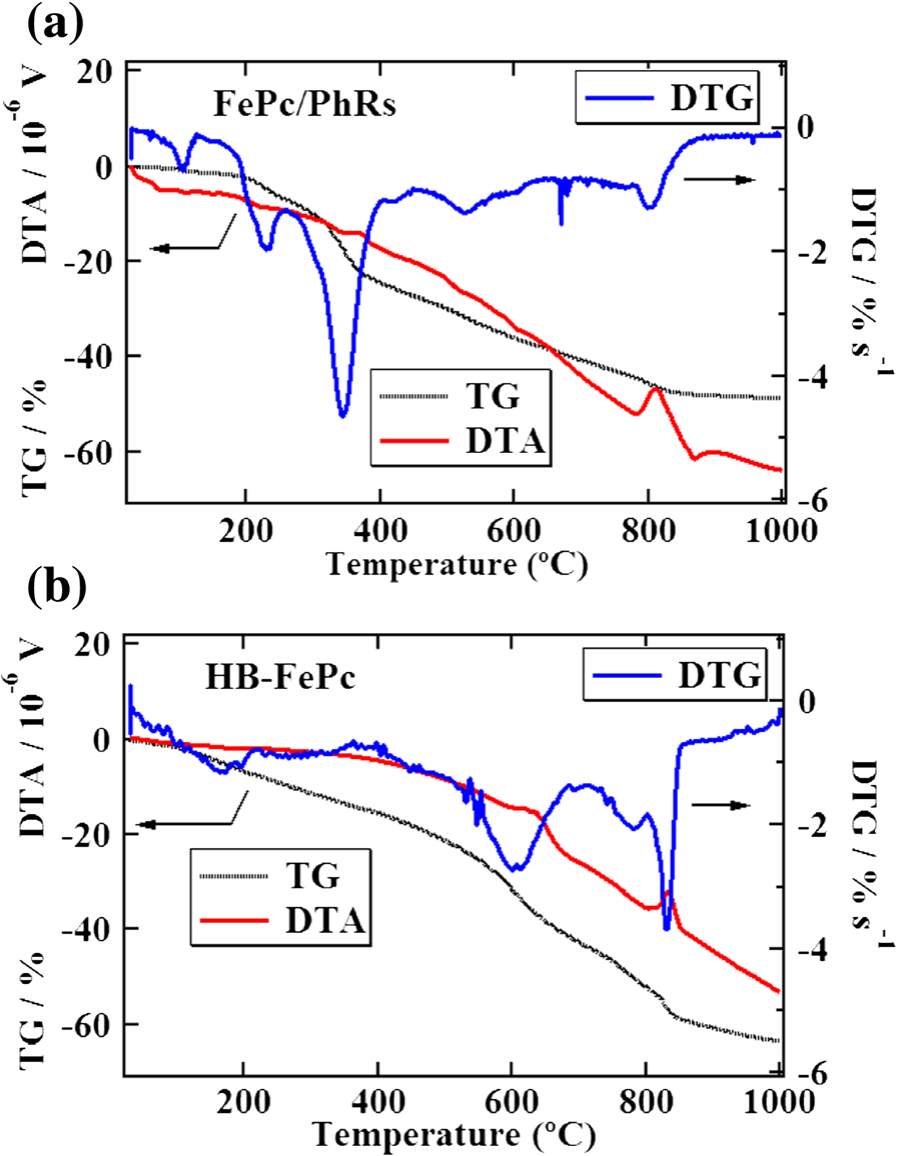
TG/DTA and DTG curves. **(a)** FePc/PhRs and **(b)** HB-FePc precursor.

TG-DTA/DTG curves of the FePc/PhRs and HB-FePc precursors are presented in Figure 3. In the case of the FePc/PhRs precursor, two large DTG mass loss peaks are observed at around 230°C and 340°C. From the mass spectrum (not shown), the molecular weight of these peaks corresponds to phenol (*m*/*z* = 94). The DTG curve of the HB-FePc precursor does not show any significant peaks, implying that the biphenyl linker is stable below 500°C. Instead, the DTG curve of the HB-FePc precursor shows two large peaks at around 600°C and 830°C. The corresponding structure also appears in the DTG curve of the FePc/PhRs precursor, but with much smaller weight loss compared with that of the HB-FePc precursor. In other words, the main decomposition region of the HB-FePc precursor appears at a higher temperature (above 600°C) than that of the FePc/PhRs precursor. This may be because in the HB-FePc catalyst, the biphenyl linker connecting phthalocyanine moieties does not decompose, which suppresses liberation/sublimation of each component below 600°C. The DTA curve of the HB-FePc precursor has exothermal peaks at 650°C and 830°C, suggesting that graphitization occurs in the same temperature range as the main decomposition of the HB-FePc precursor. In contrast, the DTA curve of the FePc/PhRs precursor shows a graphitization peak around 800°C. Taking into account both the DTA and DTG curves of the FePc/PhRs precursor, decomposition occurs gradually above 600°C, while graphitization occurs exclusively at around 800°C.

**Figure 4:**
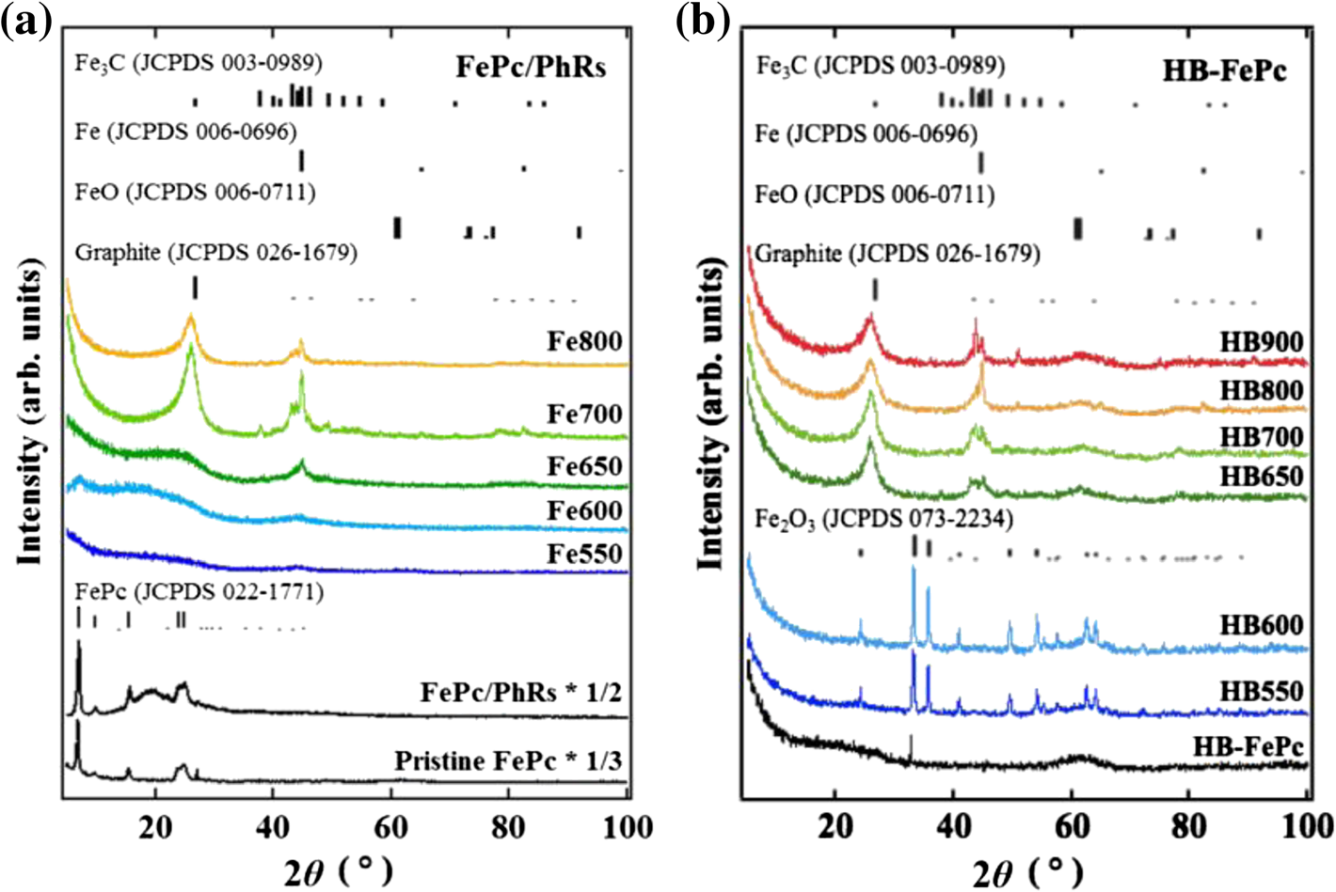
XRD patterns. **(a)** FePc/PhRs and **(b)** HB-FePc catalysts.

In the XRD patterns shown in Figure 4, quite strong diffraction peaks are observed for the pristine FePc precursor as a result of its crystalline nature [27], whereas the HB-FePc precursor does not show any diffraction peaks because it has a large, randomly linked structure that does not readily crystallize. HB550 and HB600 have amorphous signatures as a broad structure around 26° and multiple iron oxide (Fe_2_O_3_) peaks, while above HB650 graphitized carbon network around 26° as well as iron metal (Fe) and/or iron carbides (Fe_3_C) around 45° appeared. Thus the formation of the graphitic structure and the iron metal and/or iron carbides are strongly correlated. This can be understood by considering the Yarmulke mechanism; i.e., formation of a graphitic carbon shell structure around reduced metal nanoparticles [20]. In the case of the FePc/PhRs catalysts, the intensity of the diffraction peaks at both 26° and 45° gradually increases from 600°C. These peaks are absent in the XRD pattern of HB600, indicating that the HB-FePc precursor has higher thermostability than the FePc/PhRs precursor. Instead, these peaks suddenly emerge at 650°C, and then their intensities remain almost unchanged up to 900°C. Therefore, the formation of reduced iron components and their subsequent graphitization may proceed in a different manner in the FePc/PhRs and HB-FePc catalysts against pyrolysis temperature, which is consistent with the TG-DTA/DTG results. The sudden appearance of the metallic iron components at 650°C in the XRD patterns of the HB-FePc catalysts can be explained by reduction of the iron moieties, mostly iron oxide (Fe_2_O_3_). The presence of the iron oxide in HB550 and HB600 is quite in contrast to the FePc/PhRs catalysts. As already seen in the TG-DTA/DTG results, HB-FePc precursors are very reactive around 600°C. We believe that the biphenyl linker in the HB-FePc catalyst may connect phthalonitrile fragments decomposed from FePc, while the residual iron atoms dissociate and then generate clusters, which are easily oxidized in air to form iron oxides.

### C 1s HXPES

**Figure 5:**
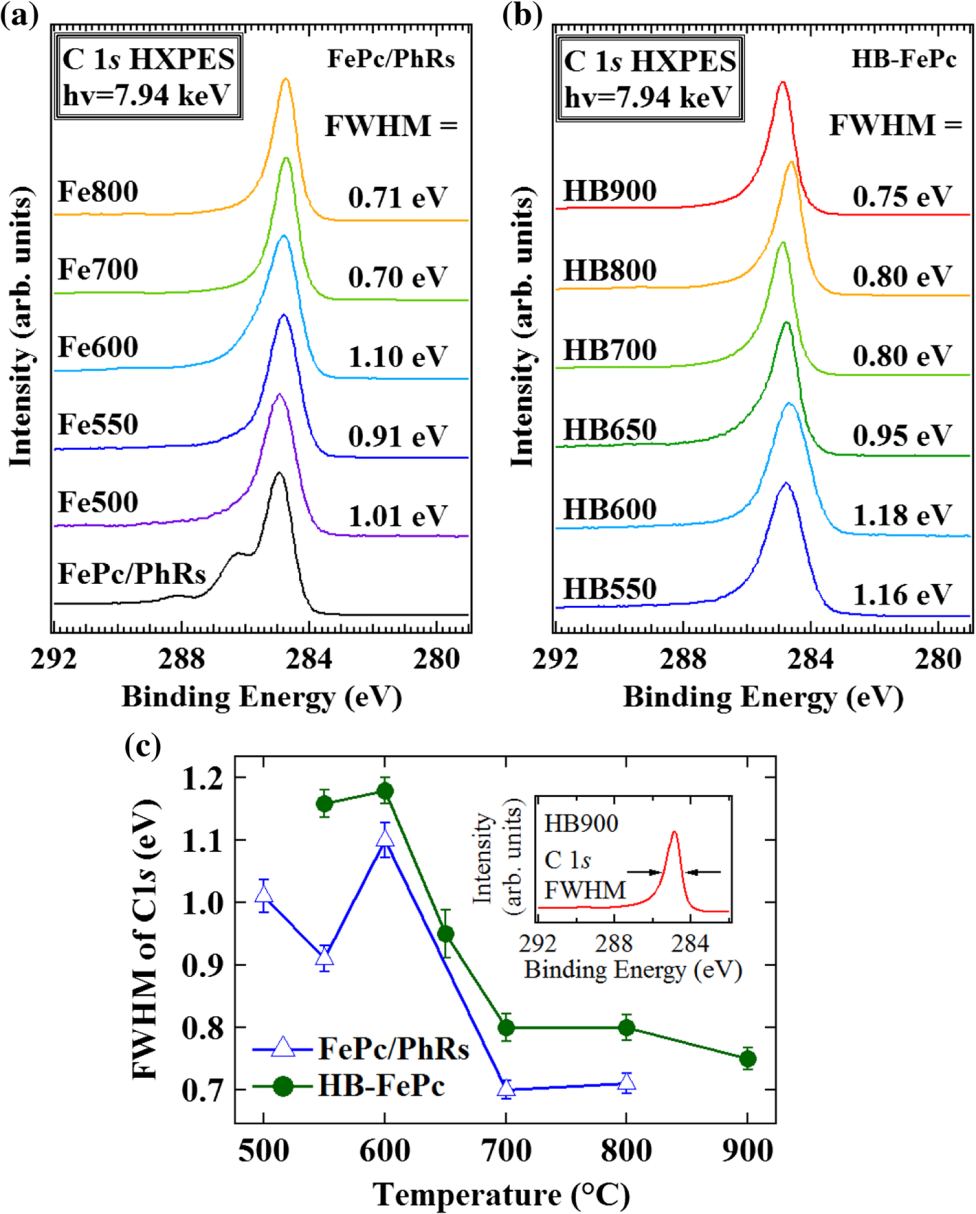
C 1*s* HXPES spectra. **(a)** FePc/PhRs and **(b)** HB-FePc catalysts. **(c)** Plot of FWHM of C 1*s* HXPES spectra as a function of pyrolysis temperature.

Figure 5a,b shows the C 1*s* HXPES spectra of the FePc/PhRs and HB-FePc catalysts, respectively. All of the pyrolyzed samples show a single peak in the range from 284.3 to 284.9 eV, indicating that the catalysts predominantly consist of typical *sp*^*2*^ carbon networks [7,10,28,29]. The FePc/PhRs precursor shows two main peaks at 284.9 and 286.2 eV corresponding to C-C and C-N bonds, respectively, and a small shake-up satellite at 288.1 eV (the same profile as in ref. [30]). The full width at half-maximum (FWHM) of the single peak was estimated by a rough fitting with a single Voigt function, which can be used to represent the degree of *sp*^2^ carbon network formation [28,29]; i.e., a smaller FWHM indicates more development of the *sp*^2^ carbon network. Figure 5c plots the FWHM of the single peak as a function of pyrolysis temperature. For the FePc/PhRs catalysts, the FWHMs increase by 0.2 eV from 550°C to 600°C, indicating decomposition of the Fe-N_4_ centers of FePc [7,14] and appearance of disordered carbon structure. In contrast, the FWHMs of the HB-FePc catalysts exhibit a very slight (0.02 eV) increase from 550°C to 600°C, suggesting that the HB-FePc catalysts do not decompose or graphitize as much as the FePc/PhRs catalysts in this temperature range because of their higher thermostability. Details of the correlation between the active site formation mechanism and carbon structure of these catalysts will be discussed in the last section.

### Fe 2p HXPES

**Figure 6:**
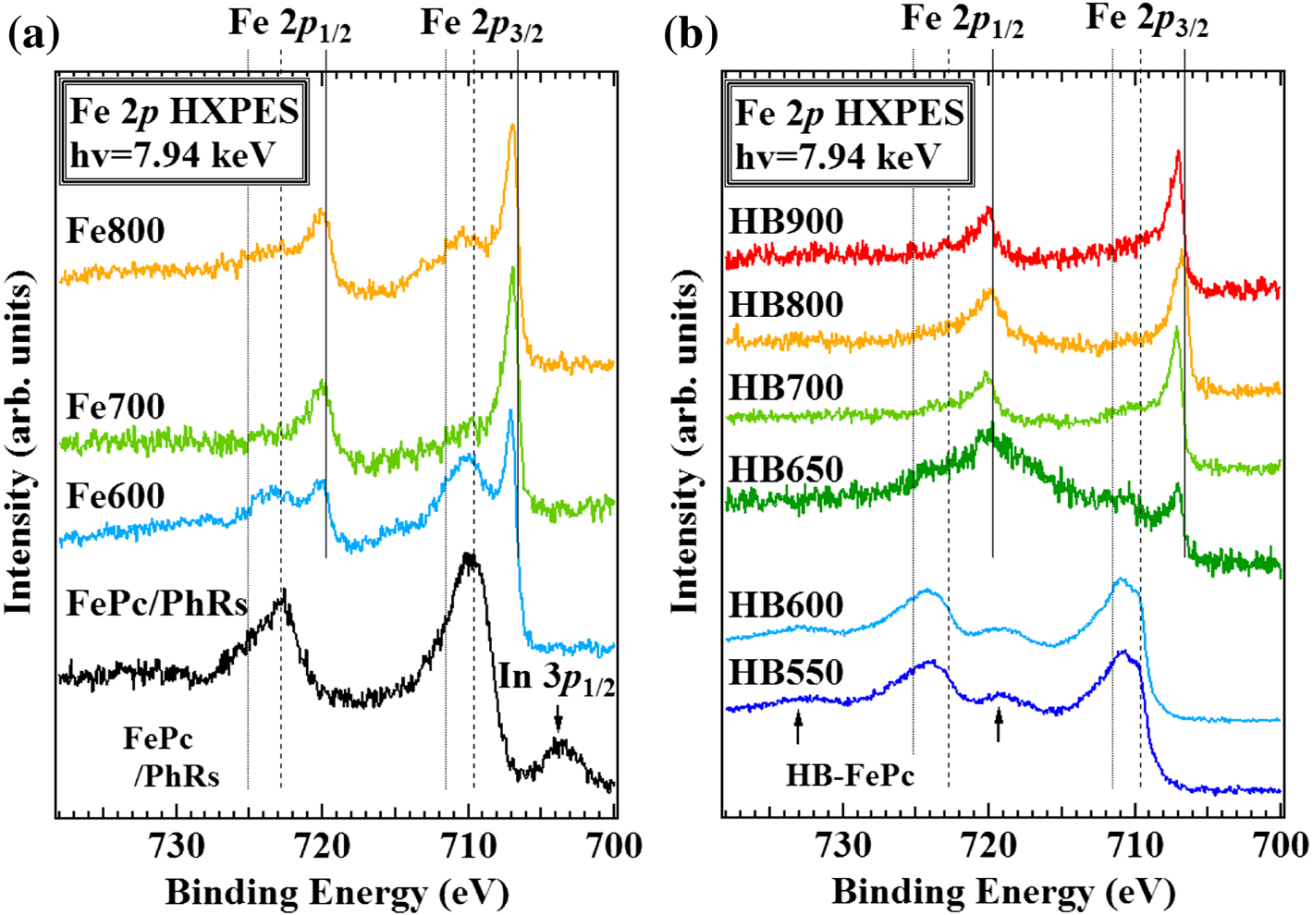
Fe 2*p* HXPES spectra. **(a)** FePc/PhRs and **(b)** HB-FePc catalysts. Solid, dashed, and dotted lines show the binding energies of Fe metal, Fe^2+^, and Fe^3+^, respectively. The arrows indicate satellite peaks of Fe_2_O_3_ 2*p*_3/2_ and 2*p*_1/2_, which emerge approximately 8 eV above pristine peaks [31]. In the spectrum of the FePc/PhRs precursor, a peak from In 3*p*_1/2_ is observed.

Fe 2*p* HXPES spectra of the FePc/PhRs and HB-FePc catalysts are illustrated in Figure 6. Solid, dashed, and dotted lines show the binding energies of Fe metal (706.5 and 719.7 eV), FeO (709.6 and 722.9 eV), and Fe_2_O_3_ (711.6 and 725.1 eV), respectively [31]. The FePc/PhRs precursor shows two peaks at 709.5 and 723.0 eV. These values are about 1 eV higher than those reported previously for FePc (708.8 eV at Fe 2*p*_3/2_) [30,32] and similar to those of FeO. However, because we did not observe Fe oxides in the XRD pattern of the FePc/PhRs precursor (see Figure 4), the 1 eV shift could be caused by a chemical interaction between PhRs and FePc. In both catalysts pyrolyzed below 600°C, the main iron components are oxidized species. The peaks in the spectrum of Fe600 correspond to a mixture of FePc and Fe metal. In contrast, the peaks in the spectra of HB550 and HB600 can be mostly assigned to Fe_2_O_3_, consistent with the XRD results as already discussed. Reduced Fe metal peaks appear in the spectra of the HB-FePc catalysts pyrolyzed at temperatures at least 50°C higher than the FePc/PhRs catalysts, in good agreement with the XRD results. In both catalysts, the oxidized moieties are completely reduced to a metallic state (Fe°) at higher pyrolysis temperatures.

### N 1s HXPES

**Figure 7:**
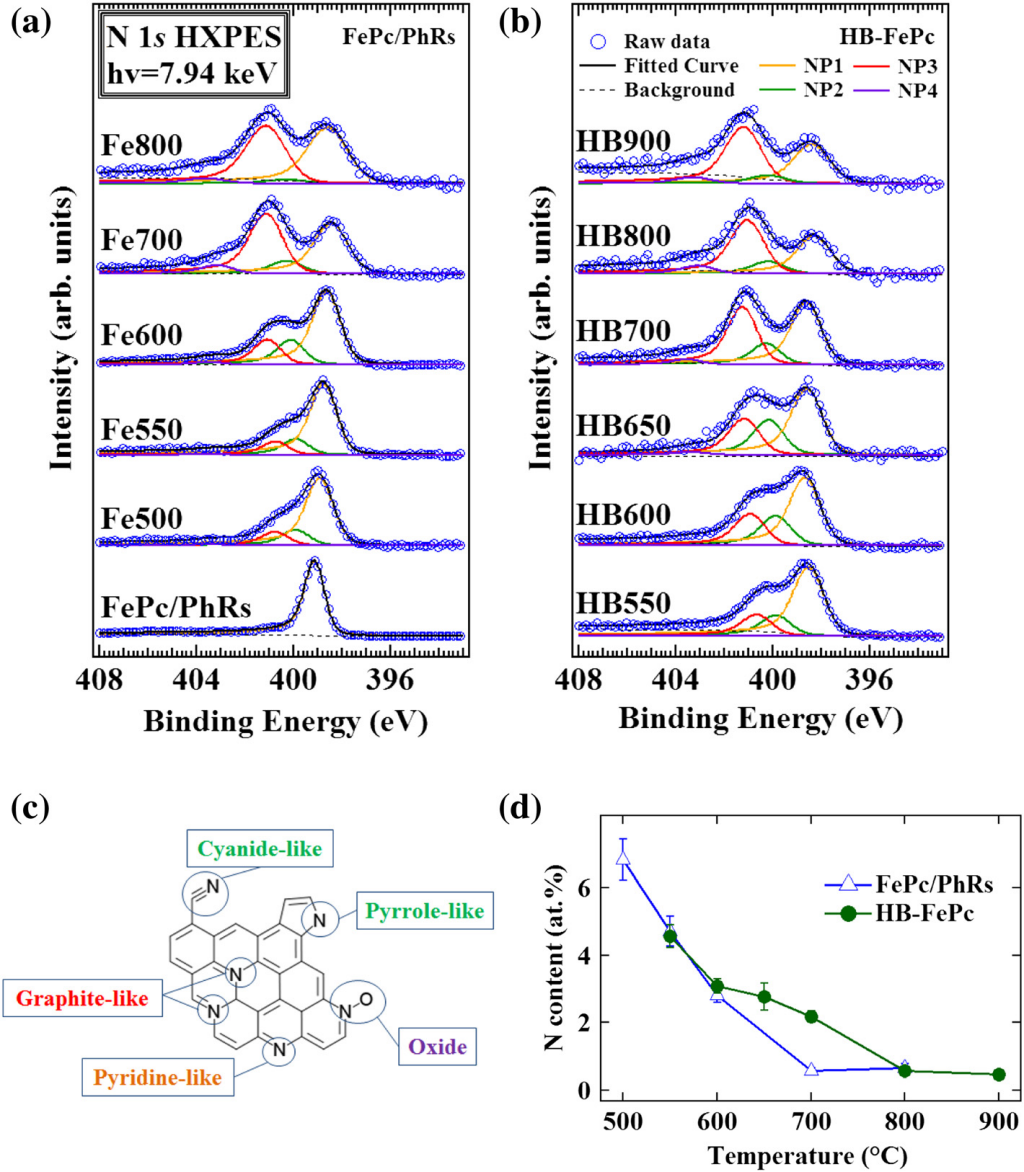
N 1*s* HXPES spectra. **(a)** FePc/PhRs and **(b)** HB-FePc catalysts, each spectrum fitted with Voigt functions followed by background subtraction by the Shirley method (dashed line). Orange, green, red, and purple solid lines are pyridine-like or FePc (NP1), pyrrole- or cyanide-like (NP2), graphite-like (NP3), and oxide (NP4) nitrogen components, respectively. **(c)** Structural formulae of four nitrogen components in graphite. **(d)** Plot of calculated nitrogen content as a function of pyrolysis temperature.

Figure 7a,b shows N 1*s* HXPES spectra of the FePc/PhRs and HB-FePc catalysts, respectively. To reveal the chemical states of nitrogen, each spectrum was fitted with a Voigt function and decomposed into different chemical species (Figure 7c). The Gaussian width was used as a common parameter among chemical species to fit the experimental data. The Lorentzian width and asymmetric parameter *α* were fixed to 0.25 eV and 0.10, respectively, where *α* is adjusted to the mean value for the C 1*s* HXPES spectra. By applying the above conditions, we were able to decompose the N 1*s* HXPES spectra into four peaks, NP1 to NP4 [7,30,33-37]. The resultant fitting parameters and relative composition ratios of the four nitrogen components of the FePc/PhRs and HB-FePc catalysts are summarized in Tables 2 and 3, respectively. The nitrogen content of FePc/PhRs was not calculated because of contamination with indium. Peak NP1 was observed at 398.2 to 398.6 eV except for FePc, where it appeared at 399.1 eV, and can be assigned to pyridine-like nitrogen (nitrogen atoms with two carbon neighbors in an aromatic ring) [7,33,35] or nitrogen of FePc [7,30]. Considering that the majority of the Fe-N_4_ structure of FePc is decomposed below 600°C as revealed by Fe 2p XPS [33], NP1 is assigned to pyridine-like nitrogen. Peak NP2 (399.8 to 400.2 eV) may be a mixture of pyrrole-like nitrogen (nitrogen atoms with two carbon neighbors in a five-membered aromatic ring) [7,33] and cyanide-like nitrogen (one neighboring carbon atom with a triple bond) [34]. Peak NP3 (400.6 to 401.1 eV) can be attributed to graphite-like nitrogen, which is nitrogen with three carbon bonds incorporated into an aromatic ring [7,35-37]. The binding energy of peak NP4 varies randomly for each catalyst (403.0 to 403.6 eV). Based on the previous reports [7,33], NP4 is assigned to N-oxide groups.

**Table 2:** **Fitting parameters and relative composition ratios of four nitrogen components in FePc/PhRs catalysts**

**Sample**	**Gaussian width (eV)**	**N content (at.%)**	**Binding energy (eV)/(composition ratio, %)**			
			**NP1**	**NP2**	**NP3**	**NP4**
Fe800	1.72	0.64	398.5	400.2	401.1	403.6
			(46.2)	(3.2)	(46.5)	(4.1)
Fe700	1.42	0.56	398.4	400.2	401.0	403.1
			(38.9)	(9.7)	(45.1)	(6.3)
Fe600	1.15	2.80	398.6	400.1	401.0	403.4
			(59.0)	(19.6)	(19.5)	(1.9)
Fe550	1.09	4.72	398.7	399.9	401.7	403.5
			(70.3)	(15.5)	(13.0)	(1.2)
Fe500	1.15	6.83	398.6	399.9	400.7	403.4
			(68.7)	(16.1)	(13.7)	(1.5)
FePc/PhRs	0.79		399.1	-	-	-
			(100.0)	(0.0)	(0.0)	(0.0)

**Table 3:** **Fitting parameters and relative composition ratios of four nitrogen components in HB-FePc catalysts**

**Sample**	**Gaussian width (eV)**	**N content (at.%)**	**Binding energy (eV)/(composition ratio, %)**			
			**NP1**	**NP2**	**NP3**	**NP4**
HB900	1.53	0.45	398.3	400.1	401.1	403.2
			(36.3)	(7.4)	(50.8)	(5.5)
HB800	1.44	0.56	398.2	400.1	401.0	403.0
			(34.4)	(11.1)	(47.6)	(7.0)
HB700	1.30	2.17	398.6	400.2	401.1	403.5
			(43.9)	(11.9)	(40.7)	(3.5)
HB650	1.30	2.76	398.5	400.1	401.1	403.3
			(47.7)	(24.8)	(25.5)	(2.0)
HB600	1.30	3.09	398.6	399.8	400.9	403.3
			(52.6)	(22.9)	(24.0)	(0.5)
HB550	1.30	4.56	398.4	399.8	400.6	403.2
			(62.1)	(18.5)	(19.0)	(0.4)

There is a small difference between the N 1*s* spectra of the two types of catalysts. The relative composition of graphite-like nitrogen (NP3) increases with pyrolysis temperature, suggesting that graphite-like nitrogen can be introduced and stabilized more easily at higher temperatures than pyridine-like nitrogen (NP1) [6,7,13]. The NP2 component is unstable and disappears at high temperatures. The composition ratio of NP4 (N-oxide group) is less than 8% (at most 0.1 at.% in total) at all pyrolysis temperatures. Taking into account that the amount of oxygen is more than 2.0 at.% in all of the catalysts, oxygen should be primarily bonded to carbon, not to nitrogen in the form of N-oxide.

### Formation mechanism of active sites

XRD and HXPES results indicate that the FePc/PhRs precursor decomposes gradually above 600°C. At the same time, thermal reduction of iron proceeds and metallic iron nanoparticles form [19]. These metallic particles accelerate the graphitization of the carbon network around them and catalyze effective nitrogen doping with considerable amounts of disordered surfaces and carbon edge structures. The XRD results support this graphitization mechanism; as shown in Figure 4, the diffraction peak of the graphitic carbon in Fe600 is quite broad, suggesting that the carbon in Fe600 mainly consists of small graphitized fractions. High ORR activity caused by the presence of such disordered, nitrogen-modified structure is consistent with the findings of other studies on carbon-based cathode catalysts derived from Fe/carbon/nitrogen precursors. Ozkan et al. reported that active carbon-based cathode catalysts have a large amount of edge planes [9,10]. Although Fe600 has a high proportion of edge structure, its degree of graphitization is insufficient [6]. Above 700°C, further graphitization occurs and stabilizes the structure, while facilitating nitrogen desorption. In fact, Figure 7d reveals that the nitrogen content of the FePc/PhRs catalysts reduces to a constant value (approximately 0.6 at.%) above 700°C. This behavior is consistent with the decreased ORR activity of the FePc/PhRs catalysts at high temperatures, which is probably caused by a decrease of catalytically active nitrogen species through nitrogen desorption. In contrast, the nitrogen contents of the HB-FePc catalysts are higher than those of the FePc/PhRs catalysts between 600°C to 800°C, as shown in Figure 7d. These observations may be associated with the thermostability of the HB-FePc catalysts where their own components are trapped more effectively than the FePc/PhRs catalysts during pyrolysis. Meanwhile, the metallic iron components effectively facilitate graphitization and trap nitrogen moieties at 650°C. Thus, nitrogen-containing fragments are presumably present when the carbon network is formed by the Yarmulke mechanism [20] in this temperature region (600°C to 800°C).

The question is why the HB-FePc catalysts do not lose their ORR activity up to 900°C even though the residual nitrogen contents above 800°C are the same (approximately 0.6 at.%, see Figure 7d) for both the FePc/PhRs and HB-FePc catalysts. We consider that the simultaneous decomposition and graphitization in the HB-FePc catalysts may effectively produce the nitrogen sites doped at the carbon edge which is responsible for the high ORR activity. Indeed, as shown in Figure 5c, HB800 has a more disordered carbon structure than Fe800 and possibly has a higher proportion of nitrogen atoms at edge sites. The role of iron in the HB catalysts seems to be the formation of the stable but disordered carbon network and the effective nitrogen uptake at high temperatures, while they do not directly catalyze ORR.

## Conclusions

Two types of carbon-based cathode catalysts were synthesized from FePc/PhRs and HB-FePc precursors, and characterized by electrochemical measurements, TG-DTA, XRD, and HXPES. The HB-FePc catalysts retained their ORR activity at high temperatures up to 900°C. TG-DTA and XRD results showed that the HB-FePc catalysts have a linked structure and high thermostability at these high temperatures. From the HXPES analysis, the FWHMs of the C 1*s* peaks of the FePc/PhRs catalysts exhibited a large increase (0.2 eV) at 600°C and then decreased above 600°C, possibly because of thermal decomposition of FePc macrocycles and subsequent graphitization. In contrast, that of the HB-FePc catalysts showed a negligible (0.02 eV) increase, so decomposition was suppressed by the biphenyl linker. In addition, the nitrogen content of the HB-FePc catalysts was higher than that of the FePc/PhRs catalysts between 600°C to 800°C. The retention of the ORR activity of the HB-FePc catalysts above 800°C may be related to the graphitization of the HB-FePc catalysts containing ORR-active nitrogen [38]. The unique active site formation mechanism in the HB-FePc catalysts provides a possibility to synthesize carbon-based cathode catalysts with improved ORR activity at high pyrolysis temperatures.

## Abbreviations

DTA: differential thermal analysis
DTG: differential thermogravimetry
FePc/PhRs: iron phthalocyanine and phenolic resin
HB-FePc: hyperbranched iron phthalocyanine polymer
HXPES: hard X-ray photoemission spectroscopy
NHE: normal hydrogen electrode
ORR: oxygen reduction reaction
PEFC: polymer electrolyte fuel cell
RDE: rotating disk electrode
TG: thermogravimetry
TM: transition metal
TMPc/PhRs: metallophthalocyanine and phenolic resin
XPS: X-ray photoemission spectroscopy
XRD: X-ray diffraction
